# Compositional Analysis of Biofilms Formed by *Staphylococcus aureus* Isolated from Food Sources

**DOI:** 10.3389/fmicb.2016.00390

**Published:** 2016-03-30

**Authors:** Elena-Alexandra Oniciuc, Nuno Cerca, Anca I. Nicolau

**Affiliations:** ^1^Faculty of Food Science and Engineering, Dunarea de Jos University of GalatiGalati, Romania; ^2^Centre of Biological Engineering, Universidade do MinhoBraga, Portugal

**Keywords:** *Staphylococcus aureus*, biofilm, food, CLSM, exopolysaccharide, protein

## Abstract

Sixteen *Staphylococcus aureus* isolates originating from foods (eight from dairy products, five from fish and fish products and three from meat and meat products) were evaluated regarding their biofilms formation ability. Six strains (E2, E6, E8, E10, E16, and E23) distinguished as strong biofilm formers, either in standard Tryptic Soy Broth or in Tryptic Soy Broth supplemented with 0.4% glucose or with 4% NaCl. The composition of the biofilms formed by these *S. aureus* strains on polystyrene surfaces was first inferred using enzymatic and chemical treatments. Later on, biofilms were characterized by confocal laser scanning microscope (CLSM). Our experiments proved that protein-based matrices are of prime importance for the structure of biofilms formed by *S. aureus* strains isolated from food sources. These biofilm matrix compositions are similar to those put into evidence for coagulase negative staphylococci. This is a new finding having in view that scientific literature mentions exopolysaccharide abundance in biofilms produced by clinical isolates and food processing environment isolates of *S. aureus*.

## Introduction

Few studies have been reported so far regarding the biofilm formation by *Staphylococcus aureus* isolated from foods (Di Ciccio et al., 2015) and the impact of the environmental factors encountered in food processing plants on the adherence and biofilm formation (Vázquez-Sánchez et al., 2013; Santos et al., 2014).

In food industry it is important to know the conditions under which *S. aureus* is able to survive, adhere to surfaces and form biofilms (Futagawa-Saito et al., 2006), leading to contamination of food products. In planktonic form, *S. aureus* does not appear resistant to disinfectants, compared to other bacteria, but it may be among the most resistant ones when is attached to a surface (Fratamico et al., 2009). *S. aureus* can produce a multilayered biofilm embedded within a glycocalix with heterogeneous protein expression throughout, forming at least two types of biofilms: *ica*-dependent, mediated by polysaccharide intercellular adhesin (PIA)/poly-N-acetyl-1,6-β-glucosamine (PNAG), and *ica*-independent, mediated by proteins (Beloin and Ghico, 2005). Biofilm-associated protein (Bap), which shows global organizational similarities to surface proteins of Gram-negative (*Pseudomonas aeruginosa* and *Salmonella enterica* serovar Typhi) and Gram-positive (*Enteroccocus faecalis*) bacteria (Cucarella et al., 2001), was the first protein that has been found to be involved in biofilm formation by staphylococcal strains isolated from mammary glands in ruminants suffering from mastitis (Speziale et al., 2014). Meanwhile, Foulston et al. (2014) discovered that the extracellular matrix of clinical *S. aureus* biofilms comprises cytoplasmic proteins that associate with the cell surface in response to decreasing pH. Regarding the capacity to form biofilms, Bridier et al. (2010) demonstrated that *S. aureus* strains from different sources (five clinical, two originating from water, two unknown, and one milk isolate from ewes with mastitis) produce biofilms with high bio volumes and high substratum coverage.

Having in view the significant damages caused by biofilms in food industry in general, more studies should be conducted to elucidate formation of such biofilms and to develop countermeasures for their removal from food contact surfaces (Marques et al., 2007). This study was carried out to evaluate the ability of *S. aureus* strains isolated from food products to form biofilms on hydrophobic surfaces at 37°C, followed by biofilm matrix characterization. The composition of the biofilms formed by *S. aureus* strains on polystyrene surfaces was first inferred using enzymatic and chemical treatments and later confirmed by confocal laser scanning microscope (CLSM).

## Materials and methods

### Bacterial strains

Sixteen *S. aureus* strains isolated from food products of animal origin (8 from dairy products, 5 from fish and fish products and 3 from meat and meat products) (Oniciuc et al., 2015) were tested to show their ability to form biofilms. Prior to inoculation, all strains were transferred from the stock cultures (preserved in 25% glycerol at −80°C) to Baird Parker (BP) (Biolife Italiana srl., Milano, Italy) and incubated aerobically at 37°C for 24 h. For biofilm assays we used overnight precultures in Tryptic Soy Broth (TSB) (Liofilchem srl., Roseto degli Abruzzi, Italy) incubated aerobically at 37°C, with shaking.

### Media screening and biofilm formation overtime

Media screening consisting in TSB with/ without addition of 0.4% glucose (TSBG) or 4% NaCl (TSBN) (Liofilchem srl.) for supporting 24 h biofilm formation was performed. Glucose (B. Braun Melsungen AG, Melsungen, Germany) sterilized by filtration (0.22 μm) was added after autoclaving. Prolonged incubation time (48, 72 h) was also performed (Peeters et al., 2007).

Biofilms were grown in 96-well plates tissue cultured (Orange Scientific, Braine-l'Alleud, Belgium) with a total volume of 200 μL of TSB, TSBG and TSBN per well and a starting inoculum approximately equal to 10^6^ CFU/mL. Only broth media were introduced in the assay as negative controls, and *S. aureus* ATCC 25923 as positive control (clinical isolate). The plates were incubated aerobically at 37°C, on an orbital shaker (ES-20/60 Environmental Shaker BIOSAN) set at 120 rpm. Biofilm quantification was performed according to the procedure developed by Stepanović et al. (2000), by using 1% crystal violet (CV) (Merck KGaA, Darmstadt, Germany). Biofilm formation in the microplates was measured in an ELISA reader set at 570 nm, and values were expressed in optical density (OD) values.

### Matrix characterization

Biofilm detachment assays were carried out as described by Kogan et al. (2006) and Fredheim et al. (2009) with slight modifications, for six strains capable to form strong biofilms with an OD>4 × OD_*NC*_. Biofilms were washed twice with 200 μL of 0.9% NaCl and then treated for 2 h at 37°C without shaking, with 200 μL of 40 mM of sodium periodate (NaIO_4_), or 200 μL proteinase K (0.1 mg/mL in 20 mM Tris-HCl:1 mM CaCl_2_). Control wells were filled with 0.9% NaCl. After treatment, the biofilms were washed once with 200 μL of 0.9% NaCl, and then resuspended into 200 μL of 0.9% NaCl and dislodged by scraping followed by sonication using a cycle of 5 s and an amplitude of 22%. Biomass quantification was performed by measuring the OD at 600 nm of each sonicated cell suspension. Measuring the OD of sonicated cell suspensions was preferred for this assay as we observed that NaIO_4_ used to assess polysaccharides reacts unspecific with CV therefore yielding false positive results.

### Biofilm composition by CLSM

The composition of 48 h biofilms was observed by CLSM, exposed to three types of dyes: (i) SYTO dye that stains nucleic acids; (ii) FilmTracer SYPRO Ruby Biofilm Matrix stain (Invitrogen, Paisley, UK), which labels most classes of proteins (Berggren et al., 2000); (iii) wheat germ agglutinin (WGA) conjugated with Oregon Green (Invitrogen), which stains *N*-acetyl-D-glucosamine residues (Wright, 1984). The fluorescence of dyes was detected using the following combination of laser excitation and emission band-pass wavelengths: 476 nm/500–520 nm for SYTO, 405 nm/655–755 nm for SYPRO and 459 nm/505–540 for WGA. After each staining step, the biofilms were gently rinsed with sterile water. The biofilm images were acquired in an Olympus^*TM*^ FluoView FV1000 confocal laser microscope and biofilms were observed using 40x water-immersion objective. The images were analyzed sequentially using two virtual channels. Three stacks of horizontal images (640 × 640 pixels) were acquired for each biofilm at different areas in the well. Two surfaces of two independent replicates were observed in each CLSM experiment.

## Results and discussions

Glucose and NaCl have been previously shown to induce biofilm formation in clinical strains of *S. aureus* (Fratamico et al., 2009). Measuring the effect of 0.4% glucose and 4% NaCl on biofilm formation enabled us to determine the conditions necessary for *S. aureus* strains isolated from food to form biofilms. For most strains, there was not a significant difference within the media used showing a small degree of variability regarding the amount of biomass produced, but overall, six strains (E2, E6, E8, E10, E16, E23; 37.5%) with OD > 0.4 were distinguished for higher biofilm formation with TSBG (Supplementary Figure 1, left graphic). As the determination of the total biomass over a specific period of time is a common practice for the characterization of biofilms and *S. aureus* biofilms are growing slowly, prolonged incubation times were used in our experiment too. Not surprisingly, quantification of biofilm proved a progressive accumulation of biomass during the analyzed time course (Supplementary Figure 1, right graphic). Based on these findings we further characterized *S. aureus* biofilms after 48 h of incubation.

In order to reveal the molecules behind biofilm accumulation, the biofilm chemical compositions were assessed by measuring the ability of NaIO_4_ or proteinase K to disperse *S. aureus* biofilms. Although both ATCC and food isolates have PNAG and proteins in the matrix, proteins prevail on PNAG, thus having a relevant role in maintaining biofilm structure. In this sense, biomass formed by *S. aureus* strains isolated from foods was reduced by 60–70% when anti-protein agents were used, while a reduction of 20–49% was obtained in the presence of the anti-polysaccharide agent (Table 1). Proteinase K treatment enhanced dispersion of Bap-positive *S. aureus* biofilms as demonstrated by Shukla and Rao (2013). The disruption effects observed on 48 h biofilms were similar for all isolates originating from food sources.

**Table 1:** **Biomass reduction of *S. aureus* biofilms when using metaperiodate or proteinase K**.

***S. aureus* strains**	**Biomass reduction, %**	
	**With NaIO4**	**With proteinase K**
E2	23 ± 10.34	71 ± 4.1
E6	34 ± 2.74	71 ± 0.74
E8	46 ± 11.07	69 ± 0.63
E10	20 ± 6.51	66 ± 3.5
E16	25 ± 0.71	64 ± 1.75
E23	49 ± 3.71	67 ± 6.05
ATCC 25923	28 ± 5.25	9 ± 1.9

Preformed biofilms were treated with NaIO4 or proteinase K for 2 h at 37°C. Control wells were filled with 0.9% NaCl. Average results ± SD of eight wells for each strain are shown. The experiments were performed in triplicate. Values of negative controls have been subtracted from the shown values.

Differences were observed in the biofilm disruption pattern when comparing results obtained for biofilms formed by *S. aureus* isolated from food sources with those developed by the clinical isolate *S. aureus* ATCC 25923, presenting a high density of cell clusters embedded in polysaccharides. At present, there are no references to composition of biofilms formed by *S. aureus* isolated from food sources. Literature mentions only biofilms produced by strains of *Staphylococcus* spp. isolated from a poultry processing plant, which have been described by Ferreira et al. (2014), as containing a significant amount of exopolysaccharides (EPS).

**Figure 1:**
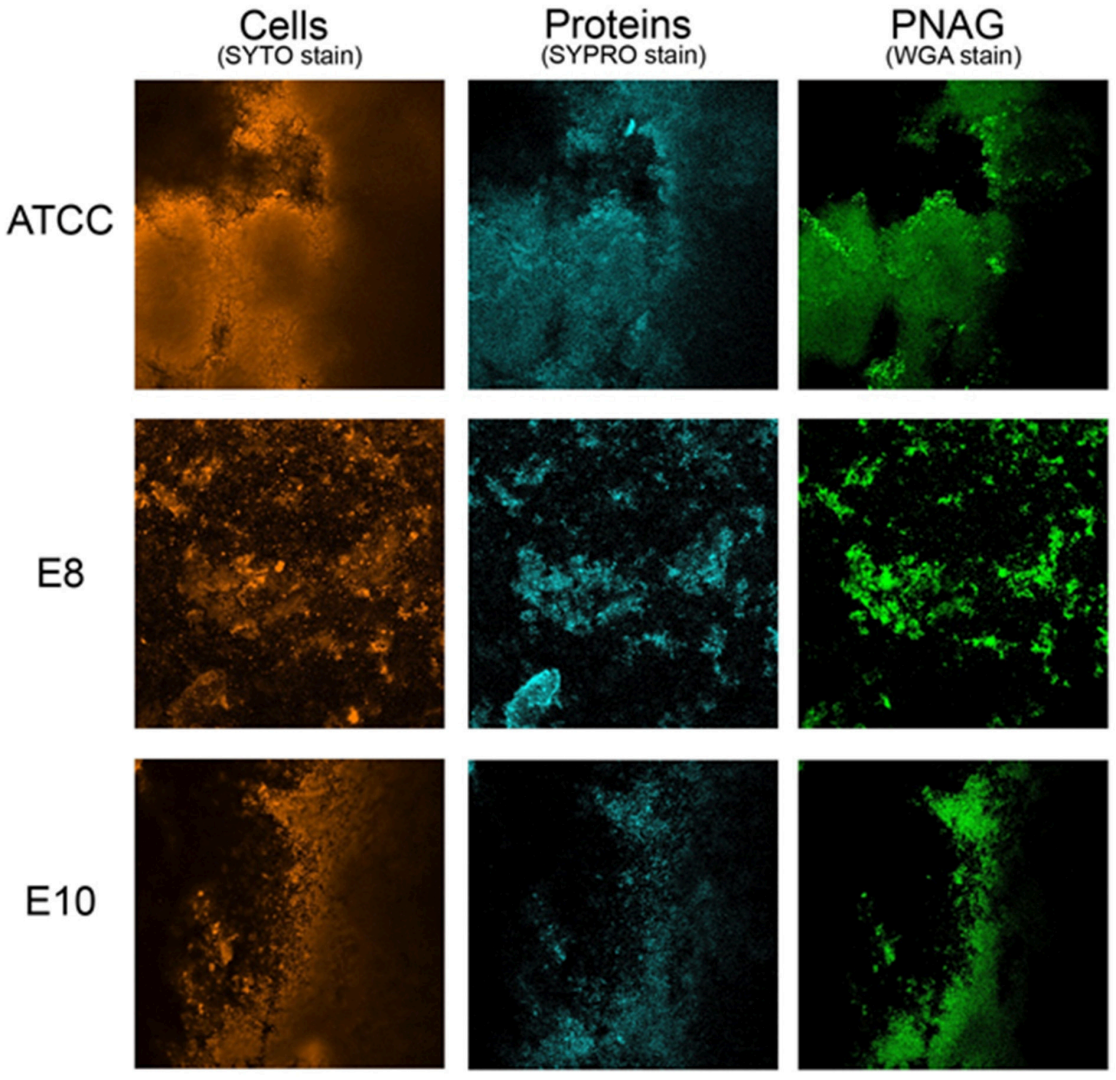
**Biofilm matrix structure obtained from confocal microscopy observations of ***S. aureus*** ATCC 25923, E8 isolated from poultry, and E10 isolated from artificial red caviar**. One z-stack is represented for each biofilm.

CLSM in conjugation with three different fluorescent dyes was used to differentiate bacterial cells from PNAG and proteins within the biofilm matrix. Qualitative approach was preferred as biofilms obtained were heterogeneous and more than three sections per each biofilm were needed for a meaningful quantification. Biofilm matrices of E8 and E10 formed by *S. aureus* strains isolated from food are represented in Figure 1 in comparison with those formed by the reference strain. These experiments confirmed that proteins are of prime importance for the structure of biofilms formed by *S. aureus* strains isolated from food sources as revealed by the quantitative approach from biofilm disruption assays.

## Conclusions and perspectives

Phenotypic production of EPS by *S. aureus* strains used in the present study suggests that staphylococcal biofilm development may have occurred *via* an *ica*-independent pathway. Clearly, in our population of bacteria, PIA independent biofilm formation was more prevalent. Nevertheless, to determine if this characteristic is in fact a key difference between food-borne *S. aureus* and clinical isolates or food processing environment isolates, future research is needed to include a broader range of food-borne isolates.

Presence of biofilm forming strains of *S. aureus* in food and food processing environments is equally important as for the medical sector. Besides causing serious engineering problems as described by Garrett et al. (2008), biofilms are involved in cross contamination events. The proteic extracellular matrix developed by *S. aureus* isolates of food origin can behave in a similar way that the one developed by clinical isolates of *S. aureus* allowing enhanced flexibility and adaptability for this bacterium in forming biofilms and supporting the formation of mixed-species biofilms either with spoilage or pathogenic bacteria as demonstrated by Foulston et al. (2014). Composition of biofilms has to be known to provide a basis for the development of better strategies for cleaning surfaces and cross contamination avoidance.

## Author contributions

All three authors contributed equally to the following sections: Introduction, Results and Discussions and Conclusions and Perspectives. EO wrote Materials and Method section together with NC and prepared the graphs shown in Supplementary Figure 1. NC prepared Figure 1. AN wrote the abstract and prepared the table. Several versions of the manuscript circulated between the authors until they all agreed on the final version.

### Conflict of interest statement

The authors declare that the research was conducted in the absence of any commercial or financial relationships that could be construed as a potential conflict of interest.

## Supplementary material

Supplementary Figure 1 ***S. aureus* biofilm development**. Biomass accumulation when using 0.4% glucose and 4% NaCl to the standard TSB (**left**). Biofilm formation overtime using TSBG (**right**). Bars represent the means of the OD value ± standard deviation (SD) evaluated in three independent measures obtained upon different treatments tested, as indicated. Values of negative controls have been subtracted from the shown values.
