# Supplementary figures and images for: Compositional Analysis of Biofilms Formed by Staphylococcus aureus Isolated from Food Sources
Source: Front Microbiol. 2016 Mar 30;7:390. doi: 10.3389/fmicb.2016.00390 (PMC4811954; doi:10.3389/fmicb.2016.00390)

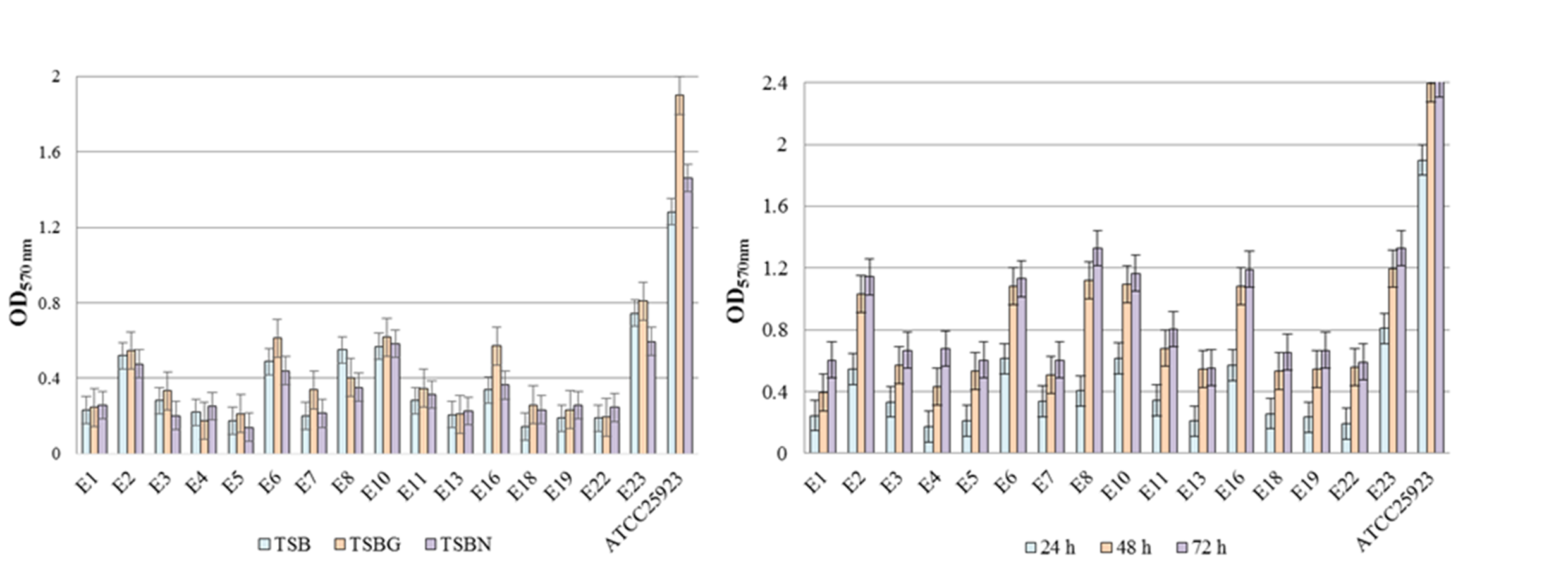

Supplement: Supplementary Figure 1 — S. aureus biofilm development. Biomass accumulation when using 0.4% glucose and 4% NaCl to the standard TSB (left). Biofilm formation overtime using TSBG (right). Bars represent the means of the OD value ± standard deviation (SD) evaluated in three independent measures obtained upon different treatments tested, as indicated. Values of negative controls have been subtracted from the shown values. [file Image1.TIF]
